# Peroxynitrite-Mediated Dimerization of 3-Nitrotyrosine: Unique Chemistry along the Spectrum of Peroxynitrite-Mediated Nitration of Tyrosine

**DOI:** 10.20900/mo.20190003

**Published:** 2019-03-06

**Authors:** Tara R. deBoer, Rafael I. Palomino, Pradip K. Mascharak

**Affiliations:** 1 Department of Bioengineering, College of Engineering, University of California, Berkeley, CA 94720, USA; 2 Department of Chemistry and Biochemistry, University of California, Santa Cruz, CA 95064, USA

**Keywords:** peroxynitrite, nitration, dimerization, inflammation, protein aggregation

## Abstract

Peroxynitrite (ONOO^−^, PN) has long been considered a potent nitrating agent implicated in numerous inflammation-mediated diseases. The current work highlights an unexplored oxidation chemistry initiated under conditions of sustained PN exposure. Impetus for this investigation developed from mass spectral results that suggested dimerization of a model peptide with a single tyrosine residue that was first nitrated following extended exposure to PN generated *in situ*. In attempts to substantiate this dimerization event and divulge the possible mode of linkage between the tyrosine derivatives of the peptide monomers, 3-nitrotyrosine (3-NT) was exposed to sustained fluxes of PN in a two-component PN-generating platform developed in this laboratory. Such exposure afforded products with tandem mass spectrometry and fluorescence spectroscopy profiles indicative of C–O coupling between 3-NT moieties. Synthesis and comparative analysis of the C–C coupled 3-NT isomer corroborated these findings. Most notably, the mass spectral data of the C–C coupled 3-NT dimer displayed a 226.80 *m/z* peak following exposure to high collision energy, corresponding to symmetric cleavage of the parent dimer peak (*m/z* = 453) along with a fragmentation product at *m/z* = 180.04 (–NO_2_ species). This fragmentation profile was distinct from the C–O coupled 3-NT dimer that exhibited a predominant 209.14 *m/z* peak with a small secondary 226.15 *m/z* peak indicative of asymmetric cleavage of the parent dimer. Results of this study indicate that formation of C–O coupled 3-NT dimer is promoted by elevated levels of 3-NT formed under high and sustained flux of PN.

## INTRODUCTION

Tyrosine nitration has remained tightly associated to the reactive nitrogen species (RNS) peroxynitrite (PN) since the discovery of PN in the early 1990’s. Early work exploited the nitration of tyrosine by PN as a strategy to define and support the physiological existence of PN. However, more recently a significant body of work has been aimed at exploring the biochemical and physiological role tyrosine nitration can play in cellular signaling, as a part of host defense, and in the onset of disease [1–4].

The fleeting nature of PN has remained a technical hurdle impeding investigation of PN-mediated nitration, where limitations in PN-production necessitate the use of chemical PN sources [5]. Under these conditions, target tyrosine-containing proteins or peptides are exposed to a burst of PN, affording a snapshot of PN-mediated nitration [6,7]. While this approach has been undoubtedly powerful in identifying proteins and enzymes that exhibit altered function or activity when one or more tyrosine residues have been nitrated, this method ceases to address conditions of chronic inflammation where PN can be continuously generated. As such, one question that has not been posed in the field of PN is, what happens after tyrosine nitration?

**Figure 1.**
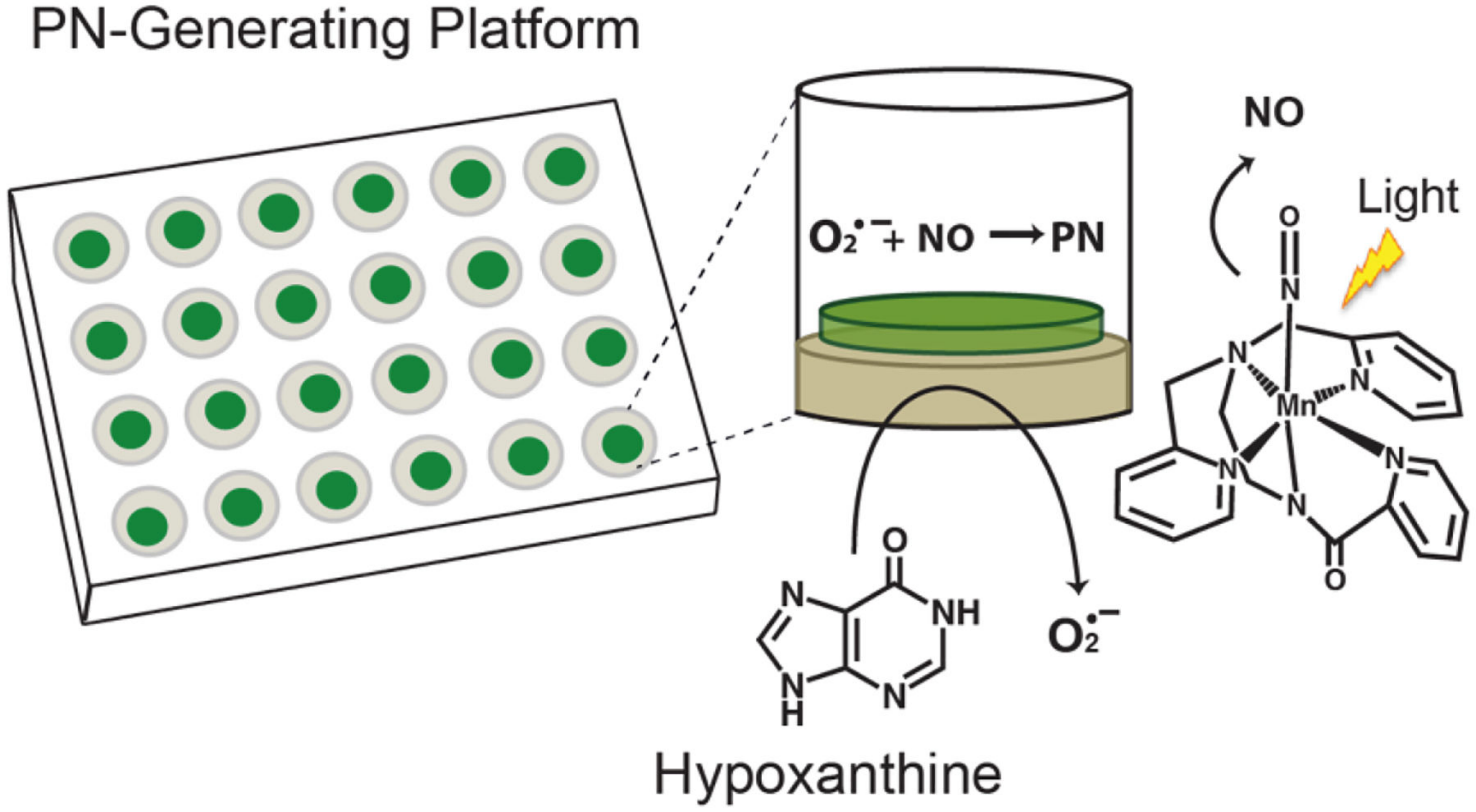
Peroxynitrite-generating platform employed to investigate the chemistry of PN. The individual wells of the platform generate PN *in situ* in a continuous and sustained fashion. The 24-well plate shown highlights the two-component system, where the O_2_^·−^-generating component is layered on the bottom of the wells and the NO-generating component is placed on top of this layer. Structure of [Mn(PaPy_3_)(NO)]^+^ is shown on the right.

We developed a PN-generating platform that mimics the enzymatic production of PN, producing continuous fluxes of precursory species nitric oxide (NO) and superoxide (O_2_^·−^) that react *in situ* to yield PN [8]. The PN-generating platform is a two-component system that is comprised of two silicate disks that have been fabricated individually and contain either photoactivatable metal nitrosyl [Mn(PaPy_3_)(NO)]ClO_4_, the NO source, or xanthine oxidase (XO) and catalase (CAT), the O_2_^·−^ source (Figure 1). When the two disks are placed together, PN formation can be initiated upon addition of hypoxanthine and exposure of the multiwell plate to a low-power visible light source, triggering the simultaneous release of NO and O_2_^·−^.

The continuous release of PN in our platform substantially extends the reaction window of PN-mediated chemistry beyond a single reaction snapshot. Previously, nitrated tyrosine (3-NT, 3-nitrotyrosine) and tyrosine dimer *o*,*o*′-dityrosine (di-tyr) were observed as the only products formed upon exposure of tyrosine to PN [9–11]. The relative formation of 3-NT and di-tyr has been reported to depend largely on the concentration of PN employed in studies, where bolus additions of chemically synthesized PN were employed. Furthermore, only under conditions of low PN and high tyrosine concentrations does di-tyr formation prevail over 3-NT [12].

Discontinuity in the continuum of tyrosine-specific modification directed by PN spurred our interest in investigating the spectrum of product evolution that can exist upon exposure of tyrosine to sustained and continuous fluxes of PN. As such, we sought to probe the breadth of PN-mediated tyrosine nitration by exploring the post-nitration chemistry of tyrosine. Herein, we report a unique coupling event between nitrated tyrosine amino acid 3-NT following continuous exposure of tyrosine to PN. Dimerization between 3-NT monomers represents an alternative unexplored chemical reaction mediated by PN.

## MATERIALS AND METHODS

### Materials

All chemicals used were analytical or ACS grade unless otherwise noted. Platform components xanthine oxidase, tetramethylorthosilicate, and hypoxanthine were purchased from Sigma-Aldrich (St. Louis, MO, USA). Catalase was procured from Spectrum Chemicals MFG Corp (New Brunswick, NJ, USA). HPLC grade solvents acetonitrile (MeCN), dimethylformamide (DMF) and trifluoroacetic acid (TFA), and 3-nitrotyrosine (3-NT) were obtained from Sigma-Aldrich (St. Louis, MO, USA).

### Synthesis of the Two-Component Peroxynitrite-Generating Platform

Peroxynitrite-generating platforms were constructed as described in our previous publication [8,13]. In short, individual NO^·^ and O_2_^·−^ generating sol-gel pieces were synthesized through the encapsulation of [Mn(PaPy_3_)(NO)]ClO_4_ (PaPy_3_ = *N*,*N*′-bis(2-pyridylmethylamine)-*N*-ethyl-2-pyridine-2-carboxamide) or XO and CAT solutions into respective tetramethylorthosilicate matrices. 0.600 mL aliquots of the metal nitrosyl sol-gel solution and 0.800 mL aliquots of the XO-CAT sol-gel were then transferred into their corresponding plates and allowed to solidify and dry in the dark for 1 week at 4 °C. Activity of the final sol-gel pieces were analyzed at random prior to use [8,13]. The individual sol-gel pieces were then combined into single wells of a 24-well plate to afford the final PN-generating system that affords ~4 μM min^−1^ fluxes of PN. Generation of NO, O_2_^·‒^, and PN was initiated with exposure of the plate to low power (10 mW/cm^2^) broadband light and the addition of hypoxanthine (final concentration 250 mM unless otherwise noted).

### Peptide Syntheses and Purification

The YTV peptide (KKYTVSINGKKITVSI) was synthesized using Fmoc synthesis on a Liberty 1 Peptide Synthesizer with Discover^®^ microwave platform (CEM, Matthews, NC, USA) on a 0.1 mmol scale, and monitored with PepDriver software [14]. Peptides were assembled on a Rink-amide ChemMatrix^®^ resin (Sigma-Aldrich, St. Louis, MO, USA). All amino acids were purchased from Aapptec (Louisville, KY, USA). The deprotection of the Fmoc group was achieved using a 0.1 M solution of hydroxybenzotriazole (HOBT) and 20% piperidine in DMF. 0.625 M diisopropylcarbodiimide (DIC) and 1.25 M HOBT in DMF were used in the coupling reactions, and all couplings were performed at 4 equivalents of Fmoc-amino acid. Each amino acid was double coupled.

A cleavage cocktail consisting of 10 mL of TFA, 0.5 mL each of 1,2-ethanedithiol (EDT) and liquefied phenol, and 1 mL of triisopropylsilane (TIPS), was added to each vessel of dried resin and reacted for 2 h followed by filtration. The filtrate was added to 90 mL of cold, dry diethyl ether for precipitation. The precipitate was collected by centrifugation, and the ether was discarded. The pellet was dissolved in 20 mL of 1:1 H_2_O/MeCN (1% formic acid) and lyophilized.

Peptides were purified by RP-HPLC on Vydac (Hesperia, CA, USA) semi-preparative C18 columns. Fractions were collected and analyzed by ESI-MS on a Micromass (Wythenshawe, UK) ZMD mass spectrometer to confirm the correct molecular weight.

### HPLC Analysis of Dimeric Products di-3NT_C–O_

Analysis of well content was carried out using a Hewett Packard 1050 Series HPLC (Palo Alto, CA, USA), equipped with a 4.6 mm × 250 mm C18 5U reverse-phase Econosphere Alltech column (Deerfield, IL, USA) and diode array detector. Samples were manually injected into a Rheodyne 7225 injection loop and eluted over 30 min with H_2_O/MeCN (both containing 0.1% TFA) at a flow rate of 0.8 mL/min. The established solvent method was as follows: 0–15 min isocratic 98:2, 15.01–20 gradient 70:30, 20.01–25 gradient 98:2, and 25.01–30 isocratic 98:2. Detection wavelengths included 220 nm, 280 nm, 310 nm and 350 nm with bandwidths of 4 nm. Chromatographic peaks were collected and lyophilized (VirTis bench top SLC lyophilizer, Gardiner, NY, USA) for fluorescence and mass spectral analysis.

### Fluorescence Analysis

Lyophilized samples were dissolved in 2 mL of 50:50 H_2_O:MeCN (0.1% formic acid) solution and analyzed by fluorescence spectroscopy (Varian Cary Eclipse, Mulgrave, Australia) with slit widths set to 10 nm. An initial excitation and emission scan was completed to define max emission and excitation bands.

### Synthesis of 3-Bromo-5-Nitrotyrosine (3-Br-5-NT)

A 50 mL round bottom flask was charged with 100 mg of 3-bromotyrosine (385 mmol) and 2 mL of neat nitric acid was added to the solid dropwise. The white solid quickly dissolved in the rapidly stirred acid and afforded a deep orange-yellow solution. To this solution, 2 mL of diethyl ether was added when a fluffy pale yellow solid separated. The solid was collected and thoroughly washed with diethyl ether. The crude solid was then recrystallized from methanol to obtain a yellow microcrystalline solid. Yield: 82%. ^1^H NMR (500 MHz, Varian Unity): 8.07 (s), 7.91 (s), 4.32 (t), 3.18 (m). IR: 3146.55 cm^−1^ (w), 1735.57 (s), 1544 cm^−1^ (s). ESI-MS (M + H)^+^: 307 and 306 *m/z*.

### Synthesis of C–C Coupled 3-NT Dimer di-3-NT_C-C_

Synthesis of the C–C coupled 3-NT dimer was achieved in reaction of the brominated nitrotyrosine derivative 3-Br-5-NT (50 mg, 163 mmol) and 3-NT (55 mg, 243 mmol) in the presence of [Cu(MeCN)_4_]BF_4_ (75 mg, 238 mmol) and Cs_2_CO_3_ (165 mg, 506 mmol) under anaerobic conditions in 20 mL of dry DMF. The reaction mixture was allowed stir for 1 h and then the solvent was evaporated down to one-third the original volume. Addition of 6–8 mL of a 50:50 methanol/ethyl acetate solution resulted in a fine powdery solid. The solid was filtered off and the filtrate was collected. Addition of dry diethyl ether to this filtrate afforded a pale yellow product. Yield: 12%. ^1^H NMR (500 MHz, Varian Unity): 8.05 (s), 8.02 (s), 7.90 (s) 7.54 (d) 7.17 (d) 4.12 (t), 3.19 (m). ESI-MS (*m/z*) = 451 [M + H]^+^.

## RESULTS

### Sustained Exposure of Model Peptide YTV to PN Yield a Post-Nitration Dimeric Product

**Figure 2.**
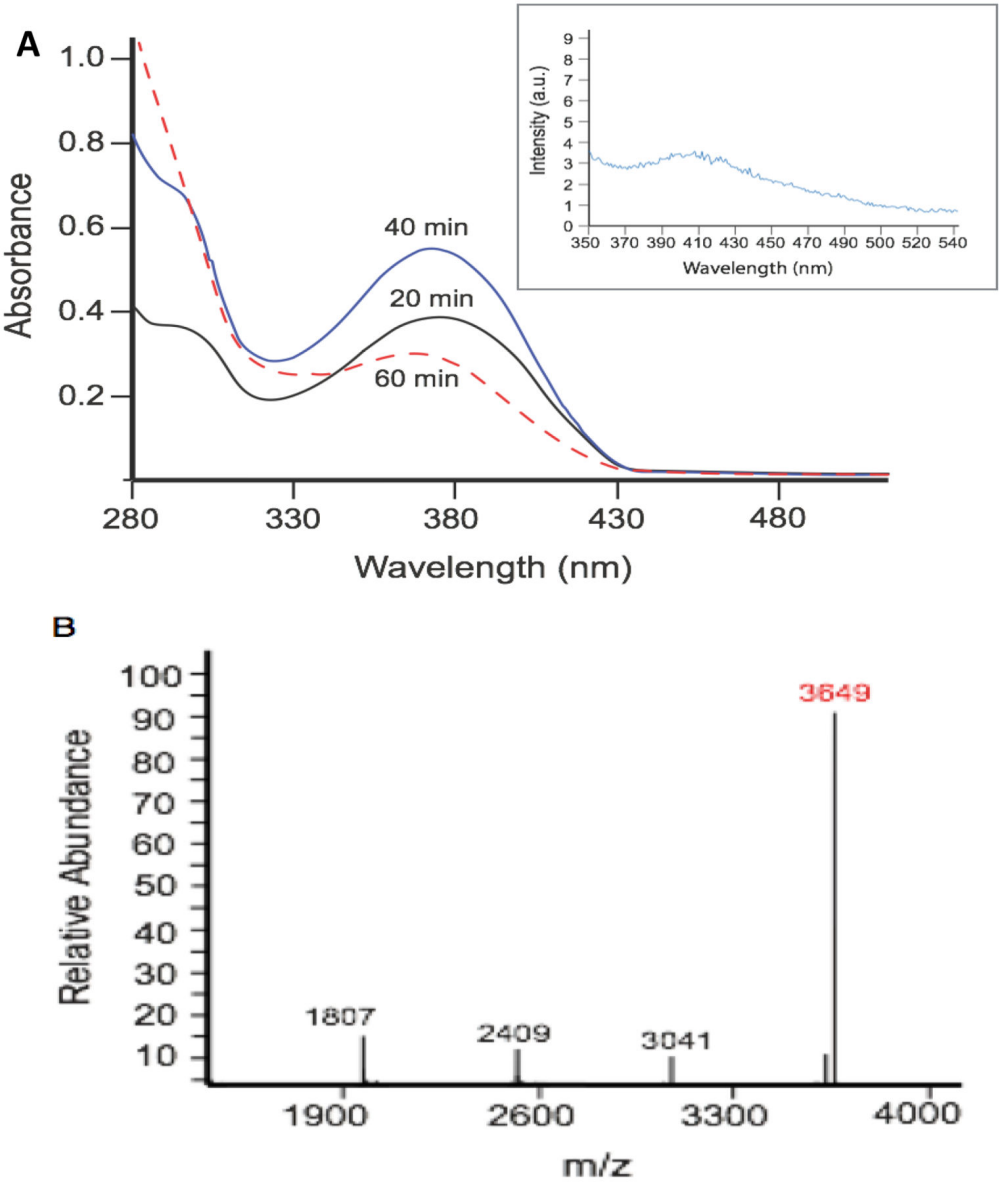
PN directs dimerization between post-nitrated YTV peptides. (**A**) Electronic absorbance spectra of the well content YTV peptide exposed to PN within the wells of the platform for 20 min (solid black line), 40 min (solid blue line), and 60 min (dotted red line). (Inset) Emission spectrum of the dimeric nitrated-YTV product, excited at 310 nm; (**B**) Deconvoluted mass spectrum of the collected YTV product, with the [M + H]^+^ peak indicative of dimerization between two nitrated YTV units highlighted in red.

In our initial efforts to capture the spectrum of chemistry observed under sustained exposure of tyrosine to PN we employed a model peptide YTV. In these studies, the PN-generating system was prepared to facilitate maximal PN chemistry, incorporating 25 mM NaHCO_3_ (4 mM final concentration) to a pH 7.4 in 0.01 M phosphate buffer solution. Under these conditions formation of reactive PN analogue nitrosoperoxocarbonate anion (ONOOCO_2_^−^) is promoted, which gives rise to strong oxidants CO_3_^·‒^ and ^·^NO_2_. The chemical transformations of YTV were analyzed after 20 min intervals of exposure to ~4 μM min^−1^ flux of PN, by electronic absorption spectroscopy. Over the course of the first two time-intervals (40 total min of PN exposure) the 370 nm absorption band, corresponding to 3-NT formation, increased as expected. However after the third interval of exposure (60 total min of PN exposure), dramatic spectral changes were observed with a sharp diminution in intensity and slight shift in the λ_max_, as highlighted in Figure 2. To explore the changes in the absorbance spectrum the well content was processed by high-pressure liquid chromatography (HPLC). Peaks from the separation were collected and further analyzed by mass spectrometry (MS). The analytical results indicate dimerization between two nitrated monomeric YTV units.

**Figure 3.**
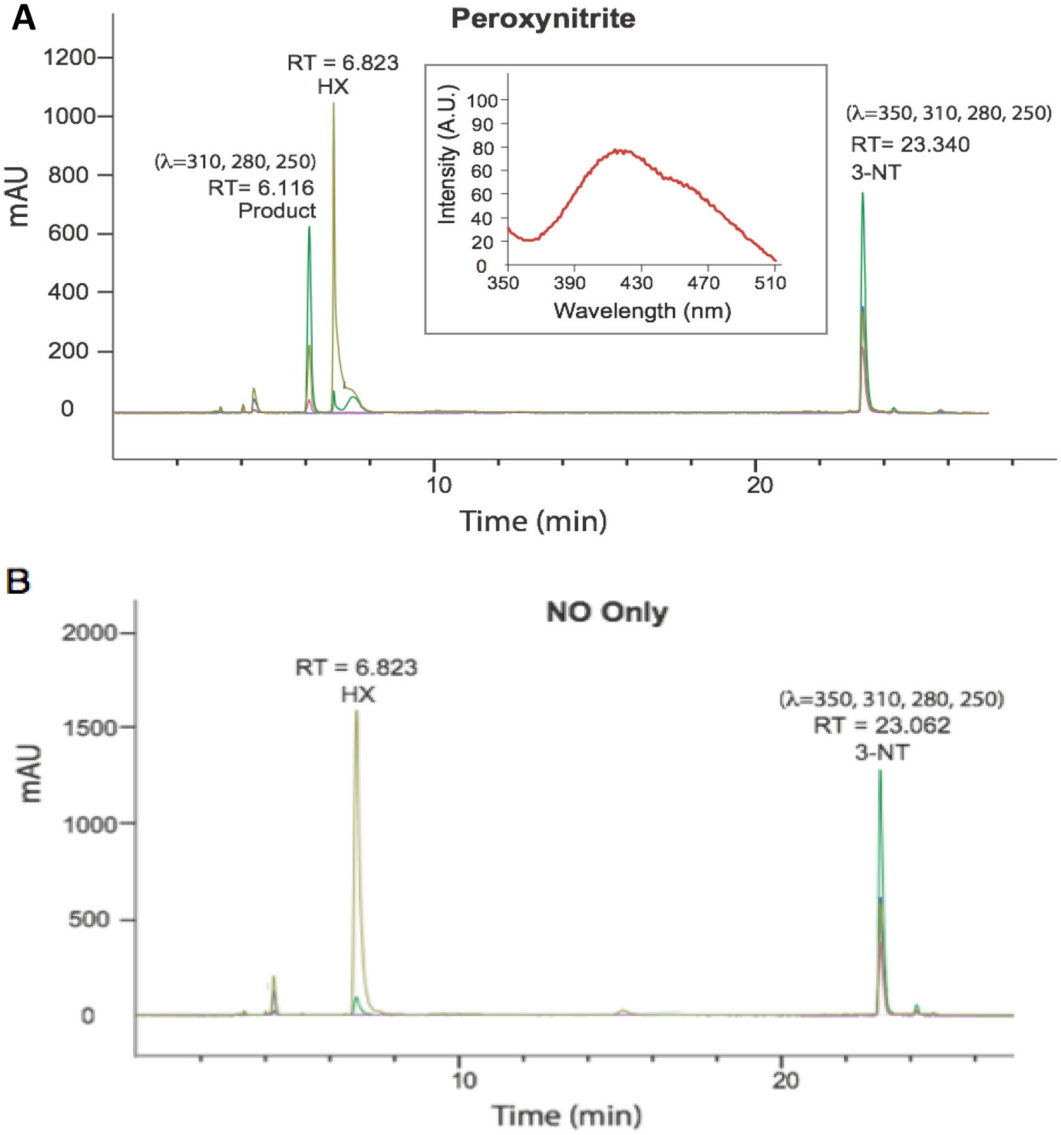
(**A**) HPLC chromatogram collected of the analyte collected from the PN-generating well content following exposure of 3-NT to PN for 30 min (Inset: fluorescence emission spectrum of the RT = 6.116 product) and (**B**) the corresponding control study completed in the presence NO only.

Dimerization was first presumed to occur with the observed [M + H]^+^ peak at 3649, corresponding to linkage between two monomer YTV units post-nitration (Figure 2B). Secondary analysis of this dimeric product by fluorescence spectroscopy featured a *biphenyl-type* emission profile, supporting coupling between aromatic residues (Figure 3A, inset) [15–17]. Given the primary sequence of the model peptide, the tyrosine residue appears to be the likely site of dimerization.

Taken together, these findings suggest that tyrosine residue within the YTV peptide is nitrated within the first 40 min of exposure to PN within the wells of the platform. We hypothesize that as the concentration of nitrated YTV peptide (YTV-NO_2_) builds in the system, the nitrated tyrosine residues become vulnerable to oxidation under sustained exposure to PN and gives rise to dimerization between YTV-NO_2_ monomer units. Therefore, to investigate the feasibility that PN mediates coupling between nitrated tyrosine residues, we proceeded to study the chemistry between PN and 3-NT.

### PN Mediated Dimerization of 3-NT Monomers

To elucidate the dimerization processes between 3-NT residues of YTV peptide monomers, we independently investigated the chemistry of 3-NT with PN. In these studies, we incubated ~160 μM of 3-NT within the PN-generating platform under the same well conditions defined in the previous YTV study, however limiting the exposure to a single 30 min interval. After 30 min of exposure the well content was analyzed by absorption spectroscopy. A hypsochromic shift in the λ_max_ from 370 to 350 nm, in concordance with the profile changes observed between the spectra of the well content of YTV experiment after treatment of 60 min of PN exposure, was observed. These changes in the absorption spectrum were also qualitatively observable, with the initial vibrant yellow color of the 3-NT solution gradually diminishing to a pale yellow hue.

The well contents were then purified and analyzed by HPLC, equipped with a multi-channel diode array detector, revealing formation of a single predominant species (Figure 3A), featuring an absorbance profile distinct from 3-NT. The product peak was detectable at 280, 250, and 310 nm but silent to 350 nm, while 3-NT shows signals over all four wavelengths. Further, the suspected product was absent when 3-NT was exposed to NO only (Figure 3B).

**Figure 4.**
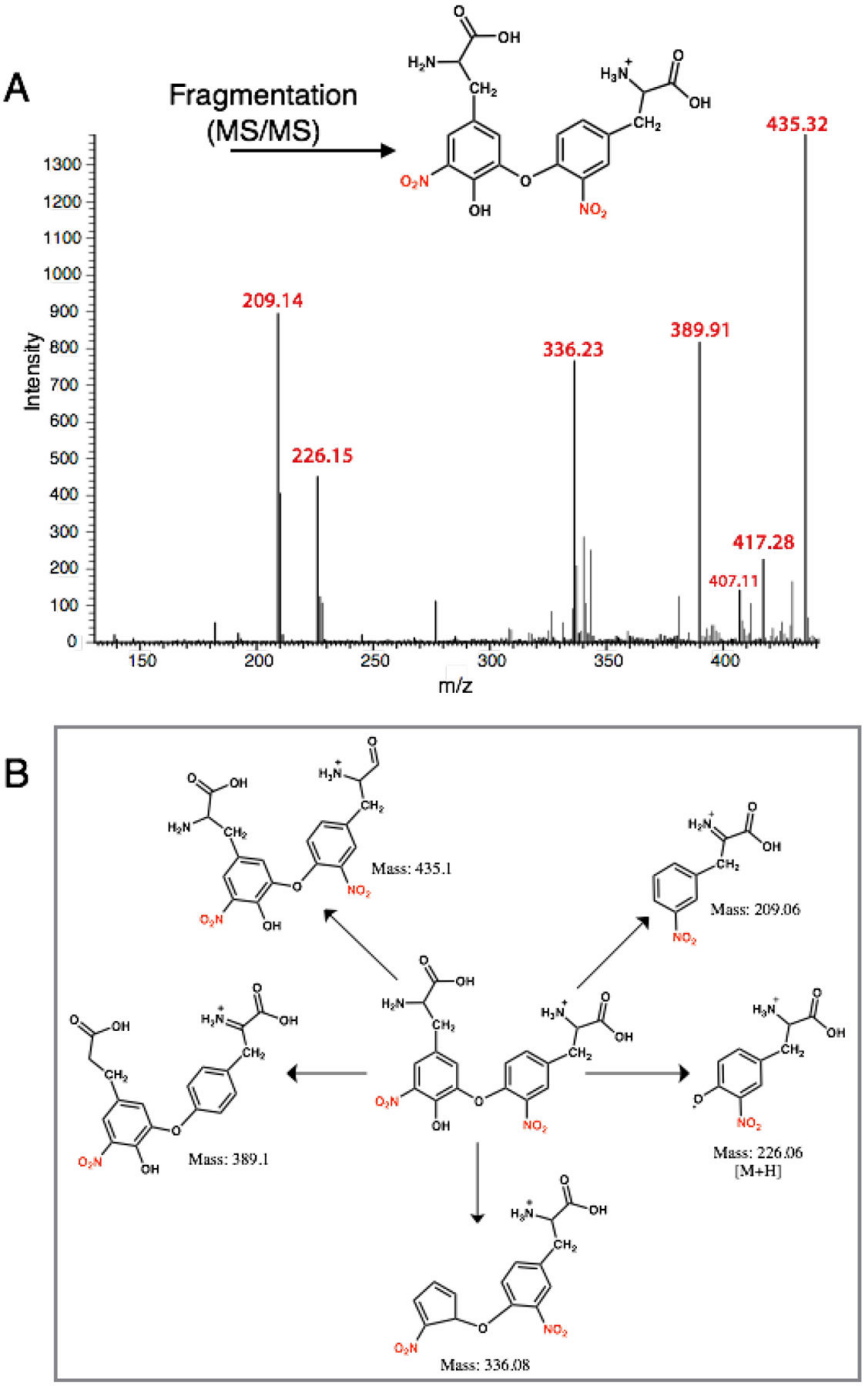
MS-MS analysis of the 3-NT coupled product (di-3NT_C–O_): (**A**) The MS of di-3NT_C–O_; (**B**) Possible fragmentation products derived from di-3NT_C–O_.

Tandem mass spectral analysis (MS-MS) of the HPLC-purified product using a LTQ-Orbitrap mass spectrometer (electron spray ionization, positive ion mode, CID) revealed *m/z* peaks at 451.40 [M + H] and 226.12 [M + 2H] confirming the formation of the 3-NT dimer (di-3NT). Fragmentation of the dimer peak (451.40 ([M + H])) required *three times* standard collision energy (60 eV) and decomposed to yield predominant peaks at *m/z* 435.32, 417.28, 389.91, 336.23, 226.15 and 209.14 (Figure 4). Peaks *m/z* 435.32, 417.28, 389.91, 336.23 correspond to consecutive −H_2_O and −HCO_2_ losses from the parent dimer and a potential rearrangement product (*m/z* = 336.23), reported previously in the fragmentation profile of 3,O′-dityrosine [18].

The predominant peaks at 226.15 *m/z* and 209.14 *m/z* suggest an asymmetric coupling event between the 3-NT monomer units, corresponding to two fragments that differ by a mass of 17 Da. Additionally, fluorescence analysis of the corresponding product featured an emission band at 420 nm with a 456 nm shoulder peak upon excitation at 268 nm (Figure 3A, inset). The profile of the emission band supports an asymmetric coupling between 3-NT units, in contrast to the reported symmetric peak profile of o,o’-dityrosine centered at 410 nm. Taken together, the results suggest 3-NT monomers asymmetrically combine upon exposure to PN to afford a C–O coupled 3-NT dimer product (di-3NT_C–O_), a coupling modality reported previously between tyrosine monomers [19].

### NT Monomers Couple via a C–O Linkage

Based on structural features inferred from the mass spectral fragmentation pattern and fluorescence emission profile we attempted to synthesize the analogous C–C coupled isomer of di-3NT (di-3NT_C–C_) to confirm the fragmentation pattern obtained in the C–O coupled product. Successful synthesis of di-3NT_C–C_ was achieved in an *Ullman type* reaction that employs a cuprous catalyst and an inorganic base to couple an activated aryl halide with a nucleophile [20]. Aryl halides are strongly activated by electron withdrawing groups that enhance electrophilicity by pulling electron density from the π-system, and the NO_2_ group represents the most strongly activating substituent [21].

**Figure 5.**
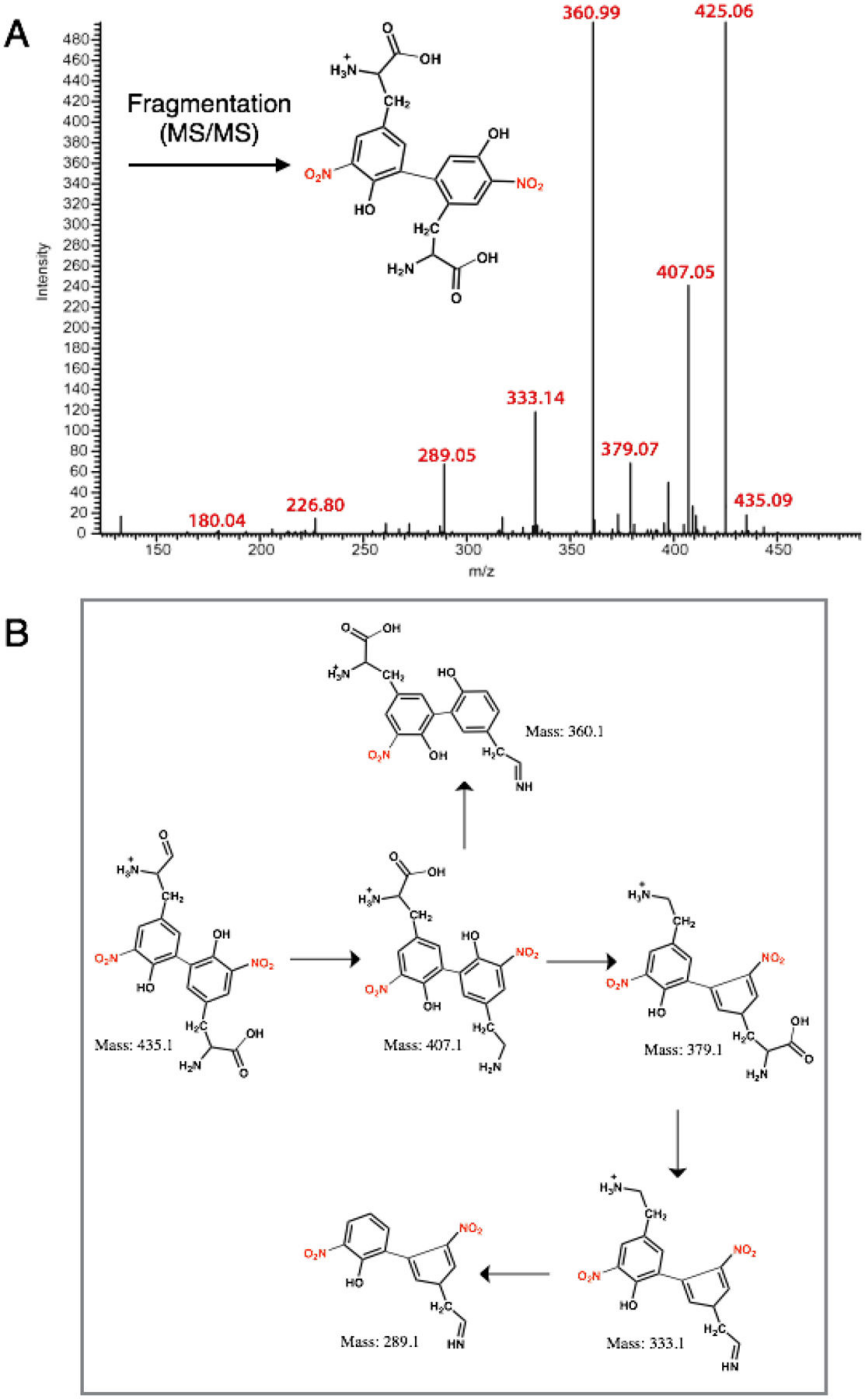
MS-MS analysis of the 3-NT coupled product (di-3NT_C–C_): (**A**) The MS of di-3NT_C–C_; (**B**) Possible fragmentation products derived from di-3NT_C–C_.

The nucleophilic aromatic substitution reaction yielded di-3NT_C-C_ upon reaction of 3-NT (nucleophile) with aryl halide 3-bromo-5-nitrotyrosine in the presence of a cuprous catalyst (Tetrakis(acetonitrile)copper(I) tetrafluoroborate, [Cu(MeCN)_4_]BF_4_) and an inorganic base Cs_2_CO_3_ in dimethyl formamide (DMF). The resulting pale yellow powder was collected and analyzed by mass spectrometry, displaying a parent dimer peak at *m/z* 451.20. Fragmentation of this peak under equivalent collision conditions yielded a fragmentation pattern (Figure 5) distinct from that of di-3NT (Figure 3B), with predominant *m/z* values at 435.09, 407.05, 379.07, 360.99, 333.14, 289.05, 226.80 and 180.40. Fragmentation peaks *m/z* 435.09, 407.05, 379.07, 333.14, and 289.05, correspond to sequential losses of −OH, −HCOOH, −CO groups fragment groups and corresponding rearrangement events that are featured in Figure 4B, and the fragmentation peaks at 226.80 *m/z* and 180.4 *m/z* correspond to a 3-NT monomer unit and the loss of a nitro (46 Da) group from this monomer unit, respectively. The loss of the nitro group from the 3-NT unit represents a characteristic feature observed even with standard 3-NT, where a predominant 181 *m/z* peak is commonly observed in a standard sample. Finally, the strong peak at 425.06 *m/z* corresponds to loss of C_2_H_2_ from the parent dimer peak at *m/z* 451.20 [18].

**Figure 6.**
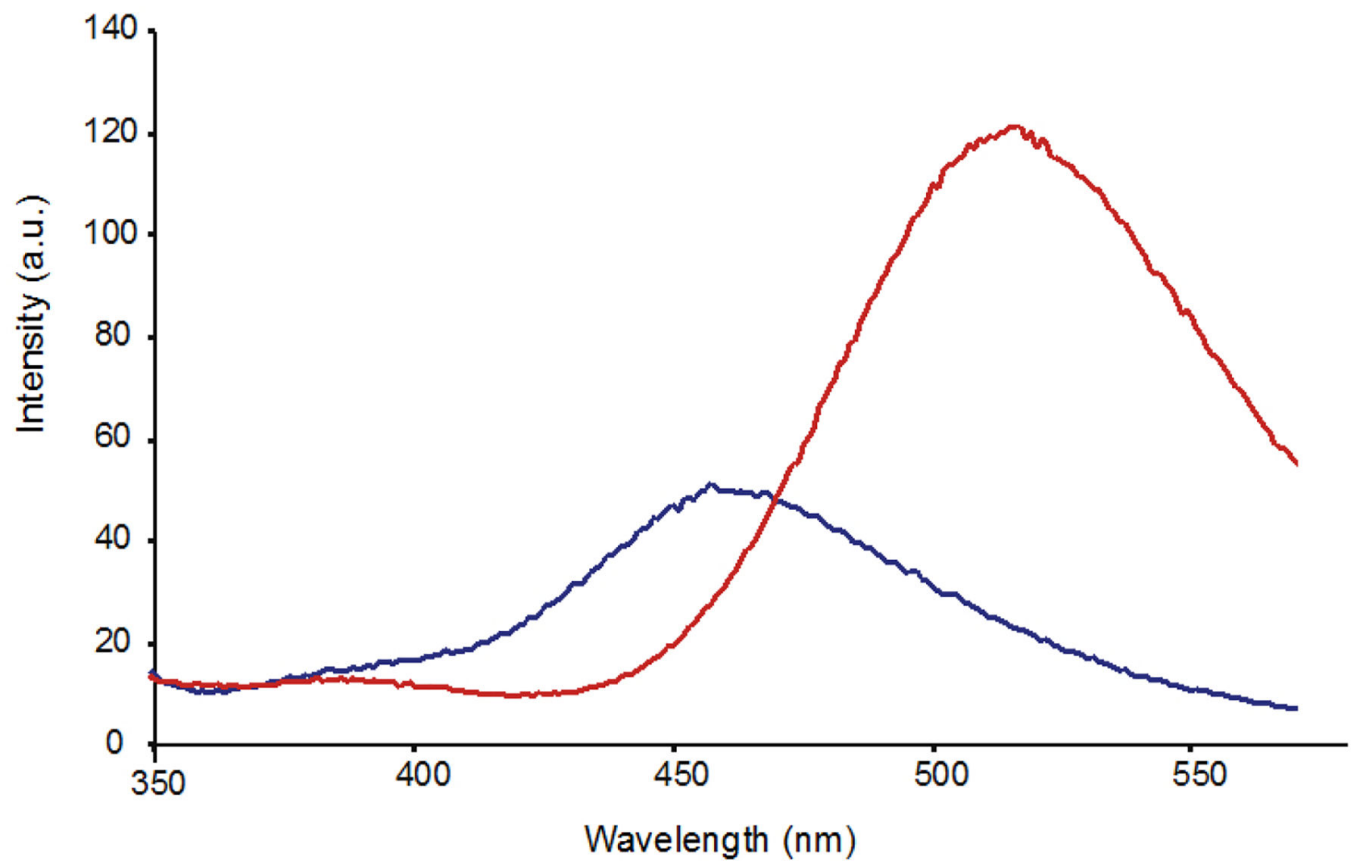
Fluorescence emission spectrum of di-3NT_C–C_ at pH 7.4 (blue line) and 10.0 (red line) upon excitation at 310 nm.

The fluorescence profile of di-3NT_C–C_ (Figure 6) features a significantly more symmetric emission band at 470 nm compared to that of the asymmetric di-3NT_C–O_ species (Figure 3A). Taken together, these spectral evidences clearly establish two different modes of coupling of two 3-NTs (di-3NT_C–C_ and di-3NT_C–O_). Comparison of the 451.2(4) fragmentation patterns of the isomers highlights a dramatic variation in the observed peak profile (Figures 4 and 5). In addition, peaks corresponding to the separation of the dimer unit into monomeric units are also distinct. Fragmentation of the proposed C–O coupled 3-NT dimer yields fragmentation products identified at 226.15 *m/z* and 209.14 *m/z* while the synthetically derived C–C coupled 3-NT dimer affords fragmentation products identified at 226.80 *m/z* and 180.4 *m/z*.

## DISCUSSION

The preceding results highlight a previously unexplored PN-mediated oxidation pathway that is initiated upon nitration of tyrosine. Post- translational nitration of tyrosine has long been the center of PN-mediated chemistry because it yields the quantifiable product 3-NT that provides a tangible marker of PN. However, the possibility that this tyrosine derivative could be further modified to form alternative products has not been explored.

At local sites of inflammation reactive species such as PN are generated for extended periods. Unfortunately, replicating such conditions chemically has proven difficult until recently. Using a two-component PN-generating platform that mimics sustained release of the reactive nitrogen species (~4 μM min^−1^) we were able to probe and monitor the evolution of PN-mediated chemistry. Initial investigation into the continuum of PN-mediated nitration was performed using a model 16-mer β-hairpin peptide YTV which contained a single tyrosine residue within the primary sequence. Continuous exposure of YTV to PN within the PN-generating platform revealed a dynamic profile of product formation, where the tyrosine nitration predominated in the first 40 min of exposure. However, as the concentration of nitrated-YTV (YTV-NO_2_) built, this species was suspected to undergo secondary oxidation that resulted in the dimerization of YTV-NO_2_ monomers.

In efforts to probe the post-nitration dimerization event between YTV-NO_2_ units, we directly investigated the chemistry between 3-NT and PN. Exposure of 3-NT to PN afforded a single predominant product that was isolated by HPLC and analyzed by absorption spectroscopy and MS-MS to be a dimeric species. Secondary mass spectral fragmentation of the 3-NT dimer suggested asymmetric coupling between 3-NT monomers, following exposure of 3-NT to PN. Comparison of the MS-MS fragmentation profile of this 3-NT dimer (di-3NT_C–O_) to synthetic C–C coupled di-3NT_C–C_ dimer corroborated the C–O linkage modality, demonstrating distinct fragmentation fingerprints.

**Figure 7.**
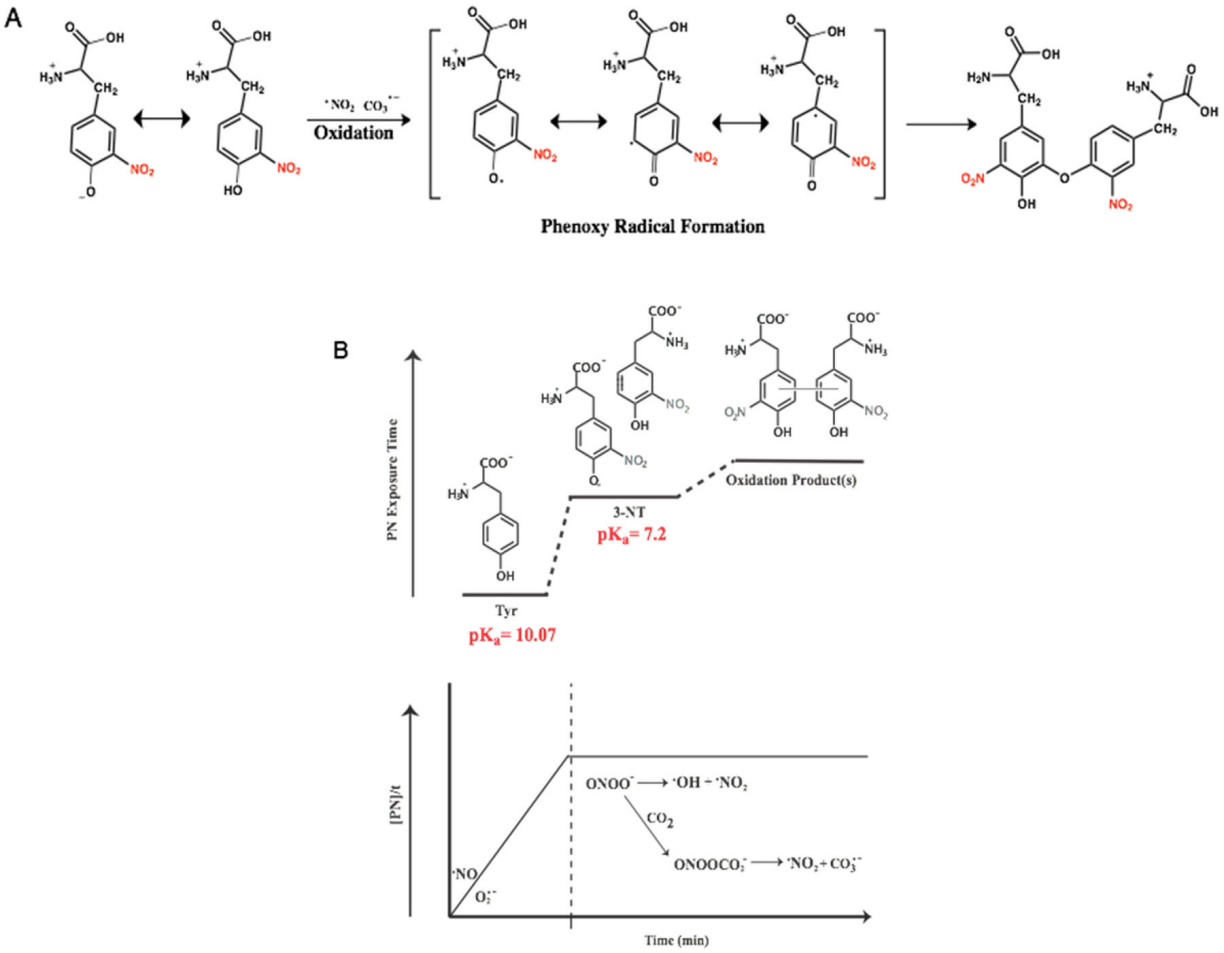
Proposed chemical profile of PN-mediated nitration tyrosine under sustained and continuous exposure to PN, where tyrosine begins as the predominant species. As the concentration of 3-NT builds, which features chemical properties unique from tyrosine, secondary dimerization species become accessible. (**A**) Radical coupling reactions lead to the formation of the C–O coupled product di-3-NT_C–O_; (**B**) Dynamic chemical spectrum of tyrosine modification mediated by PN.

Compilation of these findings supports PN-mediated oxidation of 3-NT monomers to yield di-3-NT_C–O_. Coupling between the nitrated tyrosine units most likely proceeds through the oxidation of the phenol moiety by strong oxidants CO_3_^·−^ and ^·^NO_2_ (CO_3_^·−^, E° = 1.759 V and (^·^NO_2_, E° = 1.04 V). Formation of the resulting phenoxy radical and radical dimerization events are both favored with highly substituted phenols, most commonly when a substituent is present *ortho* to the hydroxyl group. Radical coupling reactions as shown in Figure 7A then lead to the formation of the C–O coupled product di-3-NT_C–O_.

**Figure 8.**
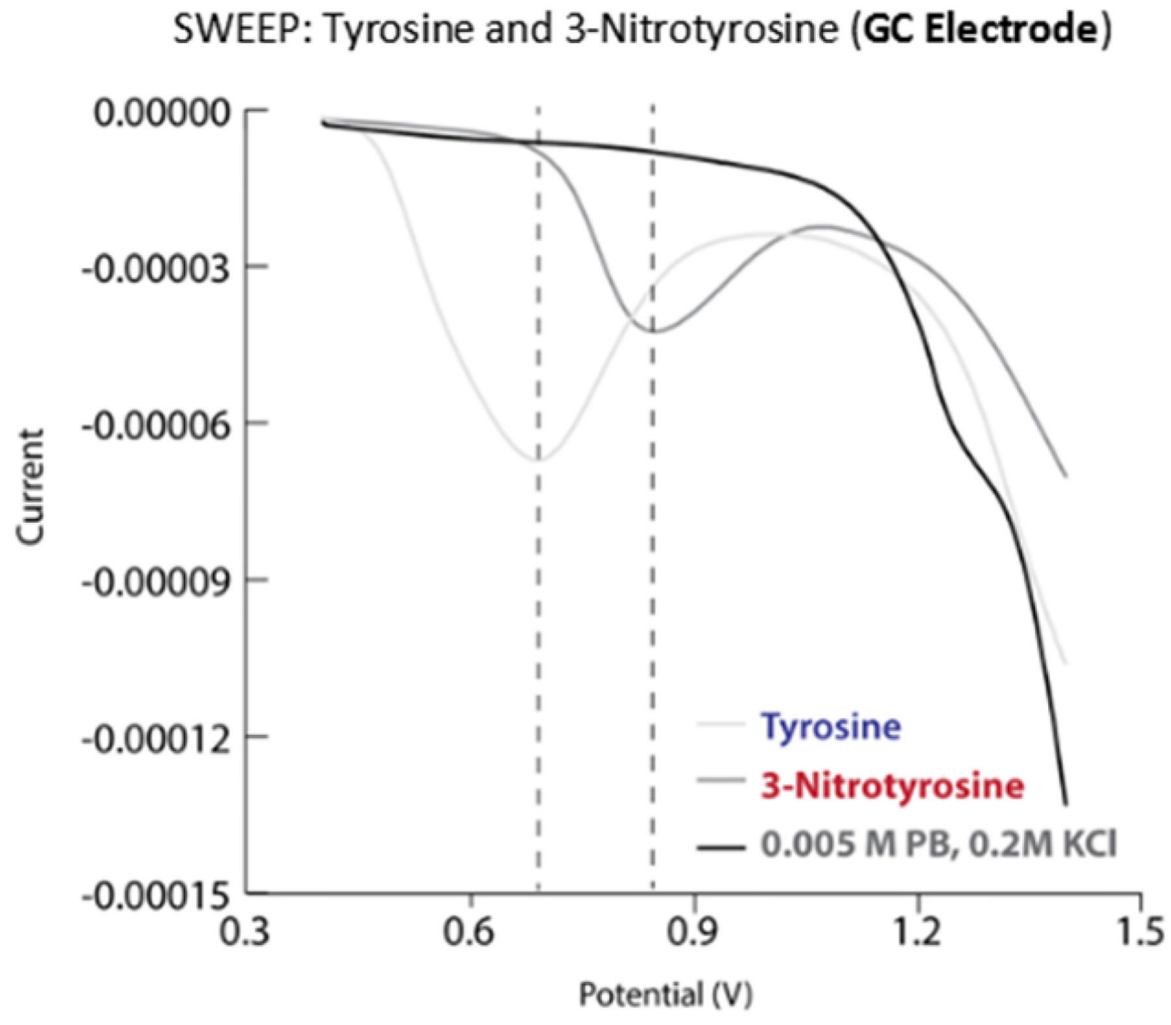
Differential pulse polarograms of tyrosine and 3-NT in phosphate buffer.

Taken together, results from the study highlight the dynamic chemical spectrum of tyrosine modification mediated by PN (Figure 7B). The continuum of PN-mediated tyrosine modification is initiated as PN directs nitration through the formation of reactive intermediate ONOOCO_2_^−^ that forms as PN reacts with CO_2_ in the system. The highly reactive ONOOCO_2_^−^ directs facile nitration of tyrosine via secondary radical species CO_3_^·−^ and ^·^NO_2_ that oxidize and nitrate the phenol moiety of tyrosine ortho to the hydroxide. Over time, the product distributions shift towards equal parts tyrosine and 3-NT, and the chemistry of the system transforms in response to the dramatic difference in chemical properties of tyrosine and 3-NT. The pK_a_ of 3-NT is 3-orders of magnitude lower than tyrosine, making it increasingly susceptible to proton-coupled electron transfer chemistry relative to tyrosine. It is important to note here that redox measurement on tyrosine and 3-NT indicate that the latter is more difficult to oxidize (Figure 8). As a consequence, oxidation of 3-NT occurs only when the 3-NT concentration reaches a critical level and the PN-flux remains relatively high as is the case in the wells of our PN-generating platform (Figure 7). In previous work, di-3NT_C–C_ has been identified as the major product at low steady-state concentrations of PN [12].

## CONCLUSION

Results of the present study reveal an alternative pathway of peptide crosslinking through C–O coupling between nitrated tyrosine residues upon *prolonged exposure to high PN flux*. This pathway could explain the formation of stable protein aggregates observed in tissues under chronic inflammatory conditions where tyrosine residues have been found to play a critical role [22,23]. Although nitration of tyrosine in protein has been considered as the predominant consequence of PN exposure, it is now apparent that 3-NT could in fact be an intermediate in the formation of an array of unsuspected and unexplored PN-mediated products. Systems capable of evolving PN for extended periods of time such as our two-component PN-generating platform are therefore essential to uncover the chemistry of this reactive nitrogen species with biomolecules.

## SIGNIFICANCE

Critical link between PN-mediated nitration of tyrosine and diseased states has now been demonstrated by the identification of this modification at critical tyrosine residues within protein and enzymes collected from *in vitro* and *in vivo* sources [1,4,7]. Although identification of the post-translational modification in collected tissue samples and cellular assays has silenced skepticism regarding the physiological relevance of this PN-mediated process, biochemical and proteomic exploration of PN pathophysiology remains at its nascent stage due to technical difficulties encountered in sustained generation of PN in laboratory settings [6]. Previous synthetic methods employed harsh chemical conditions that required rapid basification of the resulting solutions as an approach to stabilize the fleeting nature of this reactive nitrogen species. Unfortunately, this approach suffers from rapid decomposition of PN upon addition to buffered samples [5,24]. Such rapid decomposition results in minimal exposure of a biochemical system to PN. As an alternative approach, we have designed and constructed a biomimetic PN-generating multi-well platform capable of evolving high and sustained fluxes of PN [8]. PN-mediated covalent coupling has been proposed in aggregative protein α-synuclein [22,23,25]. Exposure of α-synuclein to PN yields aggregates significantly more stable than those formed following exposure of the protein to hydrogen peroxide (H_2_O_2_). Although it has been proposed that stability of α-synuclein aggregates mediated by PN is most possibly due to o,o′-dityrosine linkages (C–C coupling) between monomer units, H_2_O_2_ is also capable of imposing equivalent dimerization modalities. We suggest that additional linkages between aggregative proteins also occur through C–O linkages between nitrated tyrosine residues in the presence of PN. Comparable bond enthalpies (ΔH°) of C–C and C–O (83 and 85.5 kJ mol^−1^, respectively) infer similar relative stabilities of the C–C and C–O linkages [26]. It is quite possible that at sites of chronic inflammation, an additional (and unique) PN-mediated C–O coupling of nitrated tyrosine residues could be a cross-linking modality. Such cross-linking could lead to critical dimerization and fibril formation of aggregative proteins and provide an additional chemical feature that governs the enhanced α-synuclein aggregate stability reported previously.

## ABBREVIATIONS

PN: Peroxynitrite
RNS: Reactive nitrogen species
NO: Nitric Oxide
XO: Xanthine oxidase
CAT: Catalase
3-NT: 3-Nitrotyrosine
RP-HPLC: Reverse-phase high-pressure liquid chromatography
ESI-MS: Electrospray ionization mass spectrometry
MS-MS: Tandem mass spectrometry
di-3-NT_C–C_: C–C coupled di-3-nitrotyrosine
di-3-NT_C–O_: C–O coupled di-3-nitrotyrosine
